# Incidentally Discovered Adrenal Mass With Mild Autonomous Cortisol Secretion and Pheochromocytoma

**DOI:** 10.7759/cureus.103247

**Published:** 2026-02-08

**Authors:** Padala Ravi Kumar, Sai Madhav Reddy Duggempudi, Deepak K Dash, Debasish Patro, Samira Kumar Behera

**Affiliations:** 1 Department of Endocrinology, Maharaja Krushna Chandra Gajapati Medical College and Hospital, Berhampur, IND; 2 Department of Pathology, Maharaja Krushna Chandra Gajapati Medical College and Hospital, Berhampur, IND

**Keywords:** acth independent, adrenal incidentaloma, adrenocortical adenoma, dual secreting adrenal tumor, mild autonomous cortisol secretion, pheochromocytoma

## Abstract

Adrenal incidentalomas are adrenal masses detected on imaging performed for conditions unrelated to suspected adrenal disease. While pheochromocytomas and adrenal cortical adenomas are well-recognized entities, their coexistence with autonomous cortisol secretion (ACS) within a single lesion is rare and presents diagnostic and therapeutic challenges. A 53-year-old woman was incidentally found to have a left adrenal mass during imaging performed for evaluation of obstructive jaundice. She had hypertension but no classic symptoms of pheochromocytoma or overt Cushing’s syndrome. Adrenal-protocol non-contrast CT showed an attenuation of −13 Hounsfield units (HU), suggestive of a benign adrenal adenoma. Biochemical evaluation revealed non-suppressible cortisol on the overnight dexamethasone suppression test (ONDST) and low-dose dexamethasone suppression test (LDDST), along with suppressed adrenocorticotropic hormone (ACTH), consistent with mild autonomous cortisol secretion (MACS). Additionally, 24-hour urinary fractionated normetanephrine levels were elevated, with normal fractionated metanephrine levels, confirming catecholamine excess. Preoperatively, she was managed with alpha- and beta-blockade, followed by open left adrenalectomy. Intraoperatively, she developed hypertension requiring nitroprusside infusion, and postoperatively she experienced transient hypotension, which was managed with intravenous fluids and hydrocortisone. Histopathology revealed a collision tumor with features of adrenal cortical adenoma and pheochromocytoma. Postoperatively, her requirement for antihypertensive drugs decreased from three to one. Repeat 24-hour urinary fractionated normetanephrine levels were normal at four weeks after surgery. This case highlights the importance of comprehensive biochemical and imaging assessment in adrenal incidentalomas. The tumor’s dual-secretory nature necessitated meticulous perioperative management to prevent complications, and recognition of such rare entities is crucial for optimizing surgical and medical outcomes.

## Introduction

An adrenal incidentaloma is defined as a clinically unapparent adrenal lesion ≥1 cm in diameter detected on imaging performed for reasons other than suspected adrenal disease [1]. The prevalence in adults ranges from 1% to 6%, with higher rates in older age groups. Most adrenal incidentalomas are nonfunctional and benign, with approximately 80% being cortical adenomas [1-3]. Approximately 10%-15% of adrenal incidentalomas are functional [1,2]. Among functional tumors, mild autonomous cortisol secretion (MACS) is the most frequent abnormality (12%), followed by pheochromocytoma (7%) and primary aldosteronism (2.5%) [1-3]. The coexistence of pheochromocytoma and cortisol excess in an adrenal incidentaloma is exceedingly rare [4,5]. Cortisol excess associated with pheochromocytoma is usually caused by ectopic secretion of adrenocorticotropic hormone (ACTH) or, less frequently, by corticotropin-releasing hormone (CRH), presenting as Cushing’s syndrome [4,5]. In contrast, a benign adrenal adenoma presenting as MACS and associated pheochromocytoma is exceptionally rare [5]. To date, very few such cases have been reported in the literature [5-7].

The 2016 European Society of Endocrinology/European Network for the Study of Adrenal Tumors (ESE/ENSAT) guidelines recommended biochemical testing for pheochromocytoma in all cases of adrenal incidentaloma of size ≥1 cm [2]. However, the 2023 European Society of Endocrinology/European Network for the Study of Adrenal Tumors (ESE/ENSAT) guidelines recommended measurement of plasma free metanephrines or urinary fractionated metanephrines in all patients with adrenal lesions not typical for a benign adrenal adenoma [3]. Here, we describe a rare case of an adrenal incidentaloma cosecreting cortisol and normetanephrines, with imaging features suggestive of a benign lesion.

## Case presentation

A 53-year-old woman presented to the Endocrinology Outpatient Department (OPD) with an incidentally detected left adrenal mass identified during MRCP performed for evaluation of obstructive jaundice. She had no history of palpitations, sweating, headache, easy bruising, facial plethora, proximal muscle weakness, striae, acne, hirsutism, breast atrophy, or episodes of lower-limb weakness. There was no family history of neuroendocrine tumors or other malignancies. On examination, there were no cutaneous markers suggestive of multiple endocrine neoplasia, type 2A (MEN 2A) or multiple endocrine neoplasia, type 2B (MEN 2B). She was hypertensive, with a blood pressure of 162/92 mm Hg, without postural drop. Abdominal examination revealed no palpable mass.

Diagnostic assessment

Biochemical evaluation showed a normal complete blood count, fasting plasma glucose, two-hour postprandial plasma glucose, fasting lipid profile, liver function tests, renal function tests, and serum electrolytes (Table 1).

**Table 1:** Results of biochemical and hematological testing

Parameter	Value	Reference range
Fasting plasma glucose (mg/dL)	98	60-100
2-hour post-glucose plasma glucose (mg/dL)	138	<140
HbA1c	5.5%	4-5.6%
Serum total cholesterol (mg/dL)	188	<200
Serum LDL (mg/dL)	85	<100
Serum HDL (mg/dL)	47	Female: >50
Serum triglyceride (mg/dL)	93	<150
Serum creatinine (mg/dL)	0.7	0.6-1.1
Blood urea nitrogen (BUN) (mg/dL)	12.6	8-23
Serum sodium (mEq/L)	143	136-142
Serum potassium (mEq/L)	3.7	3.5-5.0
Bilirubin(total) (mg/dL)	0.6	0.3-1.2
Alanine aminotransferase (U/L)	23	10-40
Aspartate aminotransferase (U/L)	18	20-48
Hemoglobin (g/dL)	12.7	11-16
Total leukocyte count (cells/µL)	6073	4000-11000
Total platelet count (cells/µL)	3.5×10^5^	1.5-4×10^5^

**Figure 1:**
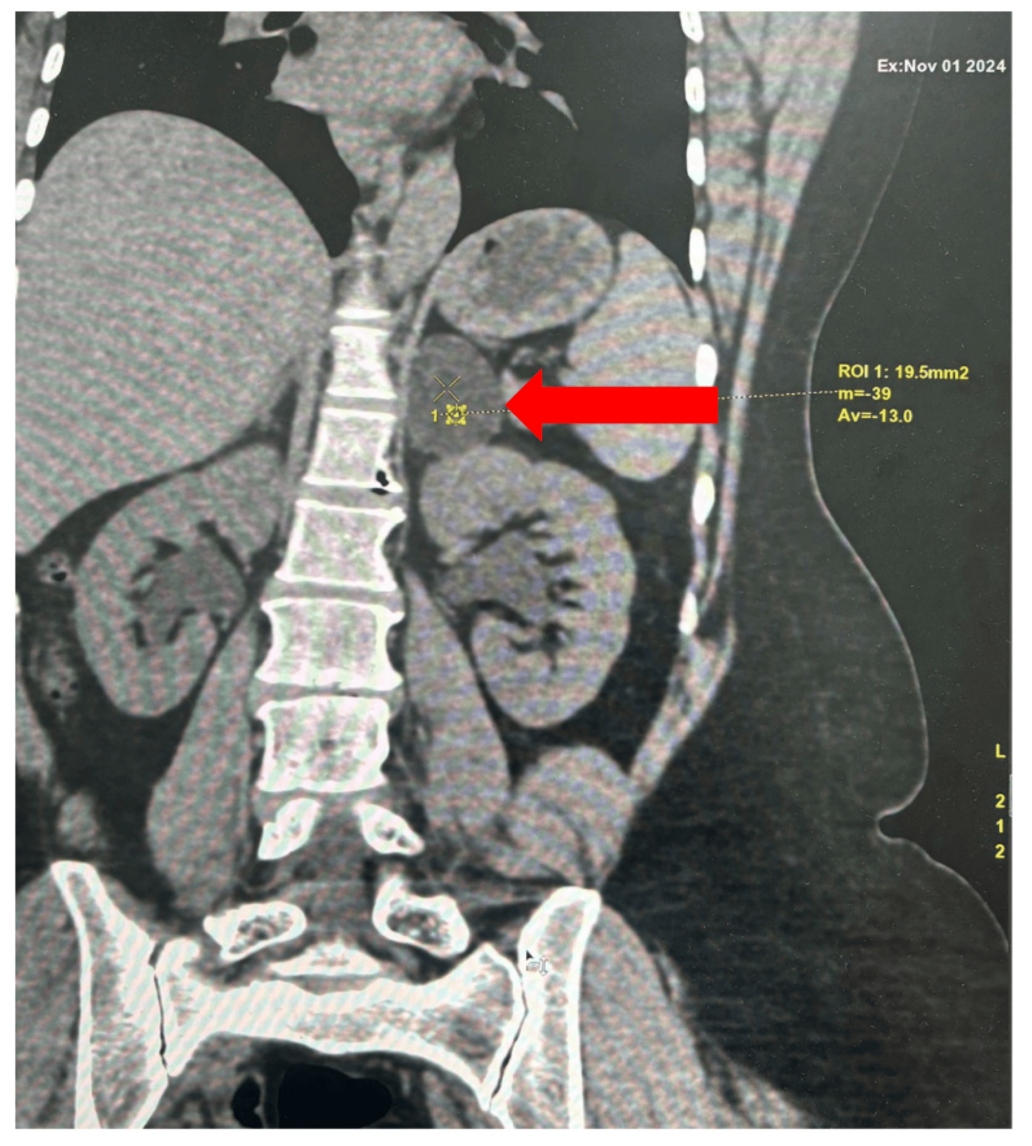
Adrenal computed tomography showing left adrenal mass of unenhanced attenuation of -13 HU (red arrow)

Thyroid function tests were normal. Abdominal CT with adrenal protocol revealed a well-defined homogeneous lesion measuring 3.6 × 3.0 × 3.7 cm in the left suprarenal gland, with unenhanced attenuation of −13 HU. Repeated and careful evaluation of the imaging could not identify any lesion with attenuation greater than 10 HU (Figure 1).

The lesion showed an absolute washout of 80%, suggestive of a benign lesion (benign lesion >60% washout), and a relative washout of 32%, suggestive of an indeterminate lesion (benign lesion >40% washout). Serum cortisol level at 8 AM was 31.88 µg/dL. Midnight awake serum cortisol at 11 PM was 9.63 µg/dL, suggestive of loss of circadian rhythm. The 1-mg overnight dexamethasone suppression test (ONDST) cortisol and low-dose dexamethasone suppression test (LDDST) cortisol levels were 9.39 µg/dL and 3.23 µg/dL, respectively. Both tests revealed non-suppressed cortisol (normal <1.8 µg/dL), suggestive of MACS. The 8 AM and 11 PM plasma ACTH values were 3.92 pg/mL and 3.01 pg/mL, respectively, suggestive of suppressed ACTH levels (Table 2).

**Table 2:** Results of dynamic testing of serum cortisol, plasma ACTH, and other hormonal assessment ONDST: overnight dexamethasone suppression test, LDDST: low-dose dexamethasone suppression test, ACTH: adrenocorticotropic hormone, DHEAS: dehydroepiandrosterone sulfate, PAC: plasma aldosterone concentration, PRA: plasma renin activity.

Parameter	Value	Reference range
Serum cortisol		
08:00 AM (µg/dL)	31.88	5–25
11 PM (awake) (µg/dL)	9.63	<7.5
Post-ONDST (µg/dL)	9.39	<1.8
Post-LDDST (µg/dL)	3.23	<1.8
Plasma ACTH		
08:00 AM (pg/mL)	3.92	10–60
11 PM (pg/mL)	3.01	5–22
Androgens		
Serum DHEAS (µg/dL)	32.30	10–193
Serum testosterone (ng/dL)	14.38	5–13
Renin–aldosterone axis		
Plasma aldosterone concentration (PAC) (ng/dL)	9.28	<40
Plasma renin activity (PRA) (ng/mL/h)	1.6	0.8–2.0
PAC-to-PRA ratio (ng/dL per ng/mL/h)	5.8	<30
24-hour urine fractionated metanephrines		
24-hour urinary metanephrine (µg/24 h)	159.36	>400
24-hour urinary normetanephrine (µg/24 h)	1328.4	>900

The 24-hour urinary fractionated normetanephrine level was 1328.4 µg/24 hours (diagnostic cutoff >900 µg/24 hours), suggestive of pheochromocytoma. However, the 24-hour urinary fractionated metanephrine level was 159.36 µg/24 hours, which was within normal limits (diagnostic cutoff >400 µg/24 hours). Serum dehydroepiandrosterone sulfate (DHEAS) was low (32.30 µg/dL). The plasma aldosterone/plasma renin activity ratio (PAC/PRA) was 5.8 ng/dL per ng/mL/h. Dual-energy X-ray absorptiometry (DEXA) revealed low bone mineral density, with a T-score of −2.8 at L1-L4, −2.2 at the right femoral neck, and −2.5 at the left femoral neck. Based on these findings, a diagnosis of pheochromocytoma with MACS and osteoporosis was made.

Treatment

For blood pressure control, oral prazosin was initiated at 5 mg and titrated to 10 mg once daily along with telmisartan (40 mg). After achieving adequate alpha-blockade, metoprolol was added at 50 mg/day. She was advised a liberal salt intake (>5 g/day). Blood pressure was well controlled before surgery. Prazosin was withheld 12 hours before surgery, and 2 L of 0.9% normal saline was administered. She underwent left adrenalectomy, and the resected specimen was sent for histopathological examination. A hydrocortisone 100 mg intravenous bolus dose was administered before surgery to cover the stress of surgery, and a hydrocortisone intravenous infusion at 4 mg/hour during surgery was given to prevent any adrenal crisis that might occur after adrenalectomy. Intraoperatively, she developed hypertension, which was managed with intravenous nitroprusside infusion. On postoperative day 1, she developed hypotension, which was managed with intravenous 0.9% normal saline. Hydrocortisone infusion was continued for 24 hours. Later, it was changed to 100 mg six-hourly from day 2 to day 7 and 50 mg eight-hourly until day 14. Later, it was changed to oral prednisolone 10 mg at 8 AM and 5 mg at 4 PM, which was tapered to prednisolone 5 mg after 1 month. Her blood pressure was under control with only telmisartan (40 mg) after surgery.

Histopathological examination

**Figure 2:**
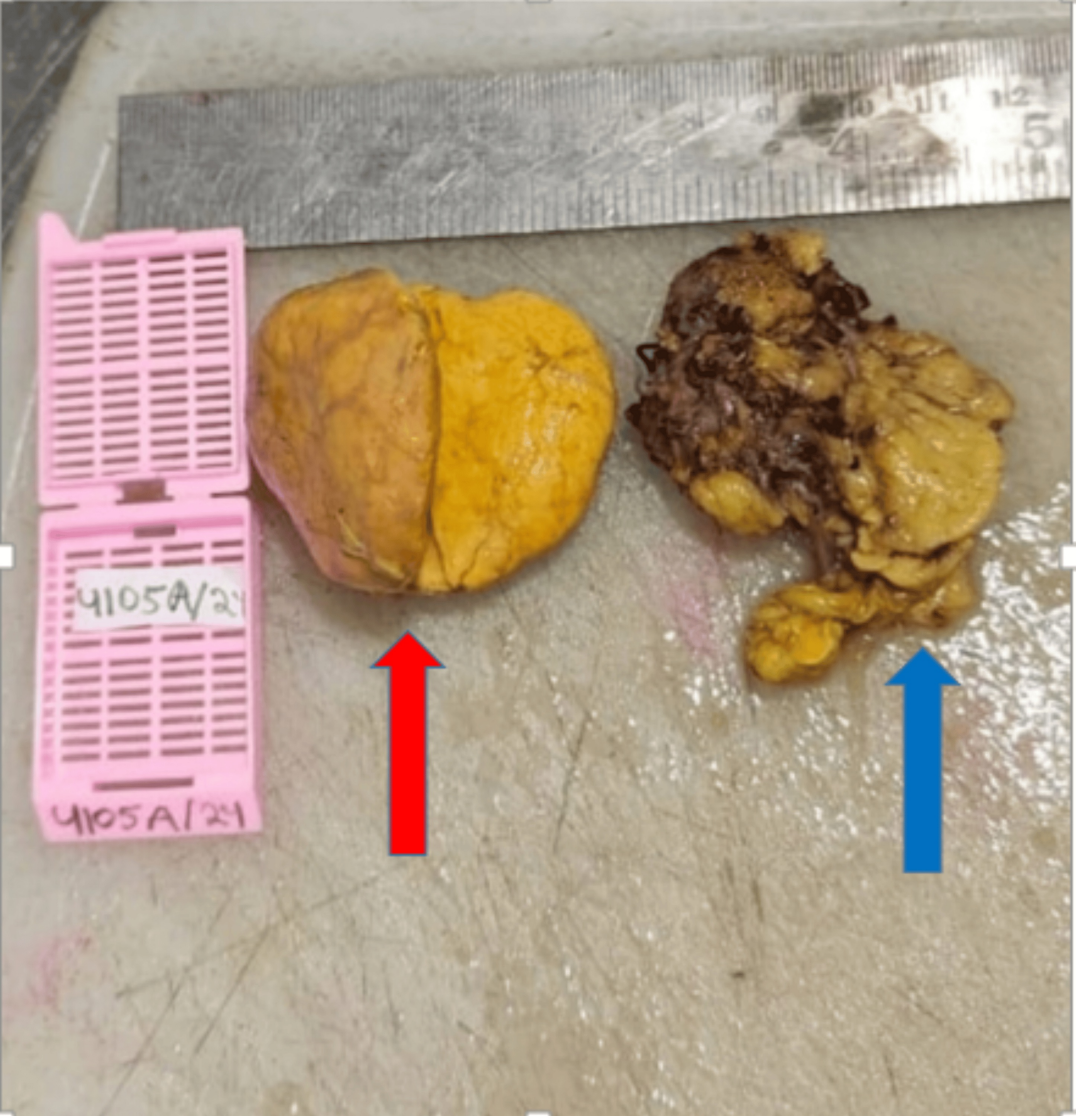
Gross image of the excised left adrenal specimen showing a nodular mass yellowish in colour (red arrow) in the adrenal gland (blue arrow).

On gross examination, the excised adrenal gland was unremarkable. A separate well-circumscribed nodular mass measuring 4 × 4 × 2 cm was identified. The cut surface of the nodular mass was solid, homogeneous, and yellowish (Figure 2).

**Figure 3:**
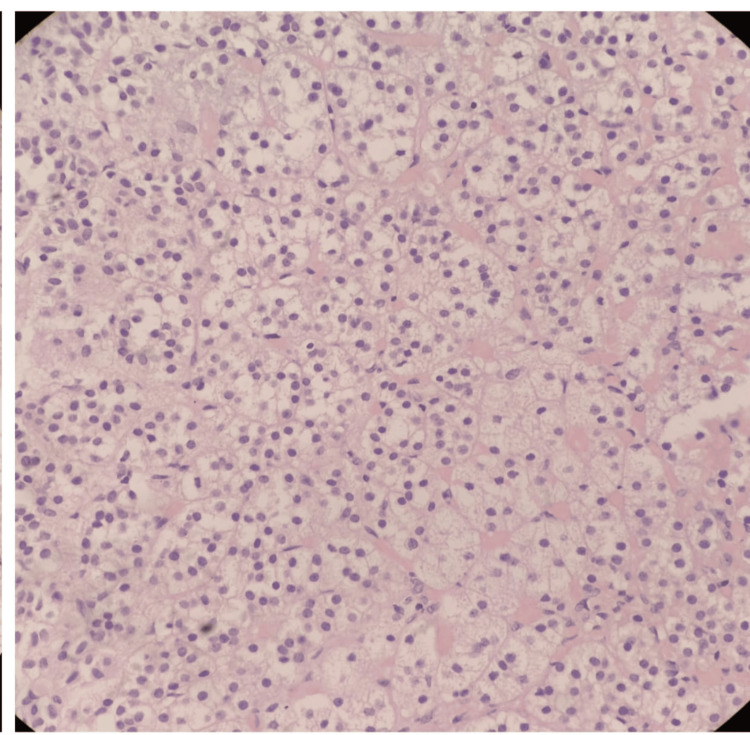
Histopathological findings of adrenocortical adenoma of adrenal tissue stained with hematoxylin and eosin (H&E) stain, showing uniform large cells with abundant foamy cytoplasm and distinct cytoplasmic borders

Histopathological examination of the nodular mass along with the adrenal gland was performed using hematoxylin and eosin staining. Microscopically, the nodular mass was composed of uniform large cells with abundant foamy cytoplasm and distinct cytoplasmic borders. No nuclear atypia, mitoses, or necrosis were identified (Figure 3).

**Figure 4:**
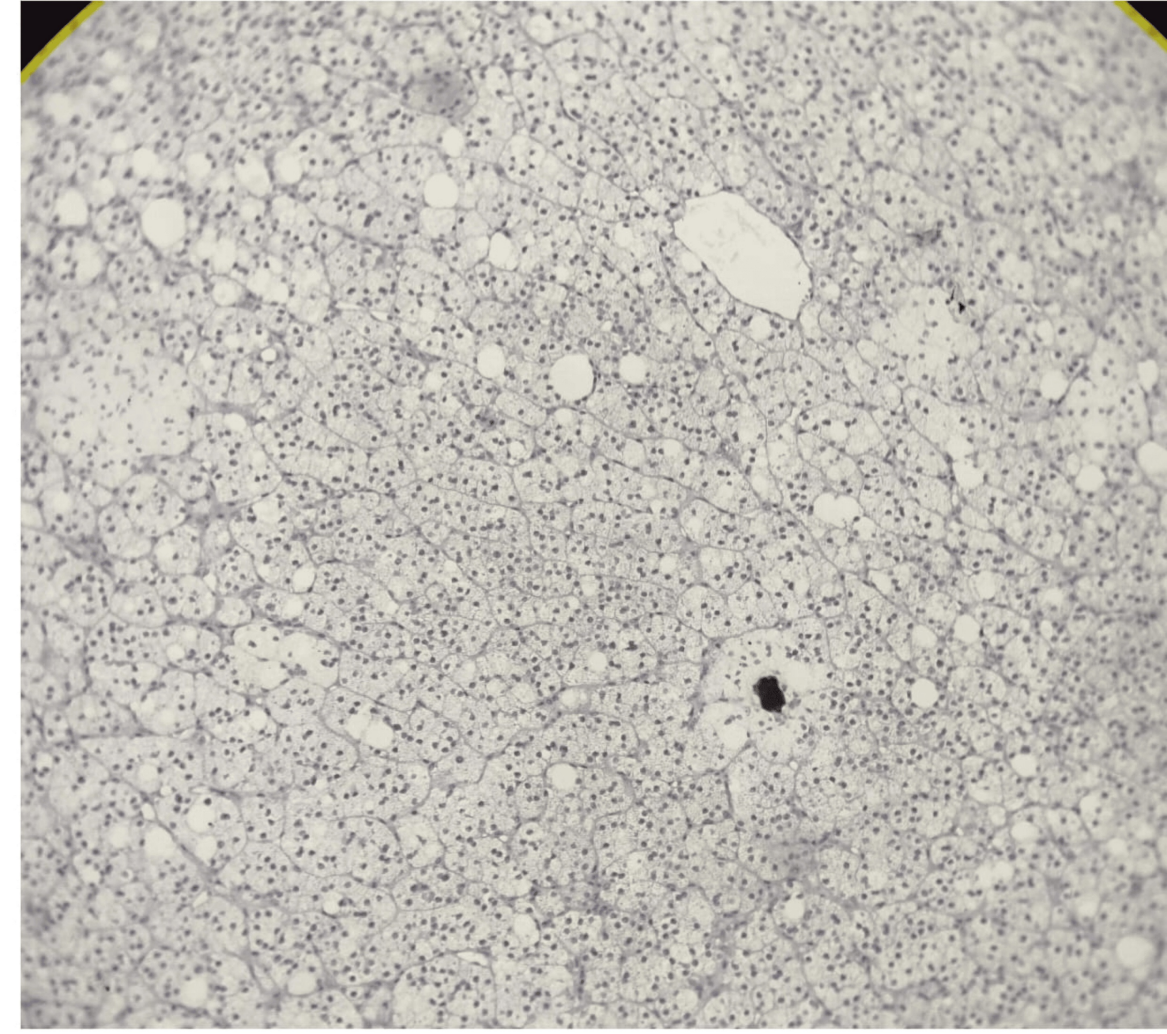
Neoplastic cells negative for chromogranin A in adrenocortical adenoma

Tumor cells were negative for chromogranin A. These features are suggestive of adrenal adenoma (Figure 4).

**Figure 5:**
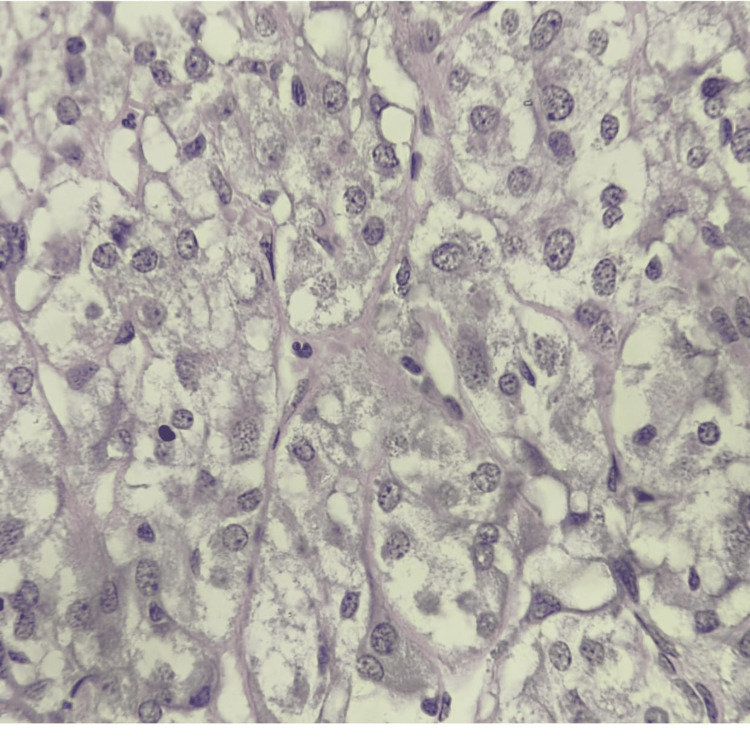
Histopathological findings of pheochromocytoma of adrenal tissue stained with hematoxylin and eosin (H&E) stain, showing tumor cells arranged in a zellballen pattern outlined by sustentacular cells

Other sections from the adrenal gland showed another focus of tumor tissue. The tumor cells were arranged in a zellballen pattern outlined by sustentacular cells. Individual cells were large and polygonal, with abundant fine basophilic granular cytoplasm and a salt-and-pepper appearance of chromatin. Nuclear atypia, mitoses, and necrosis were not identified (Figure 5).

**Figure 6:**
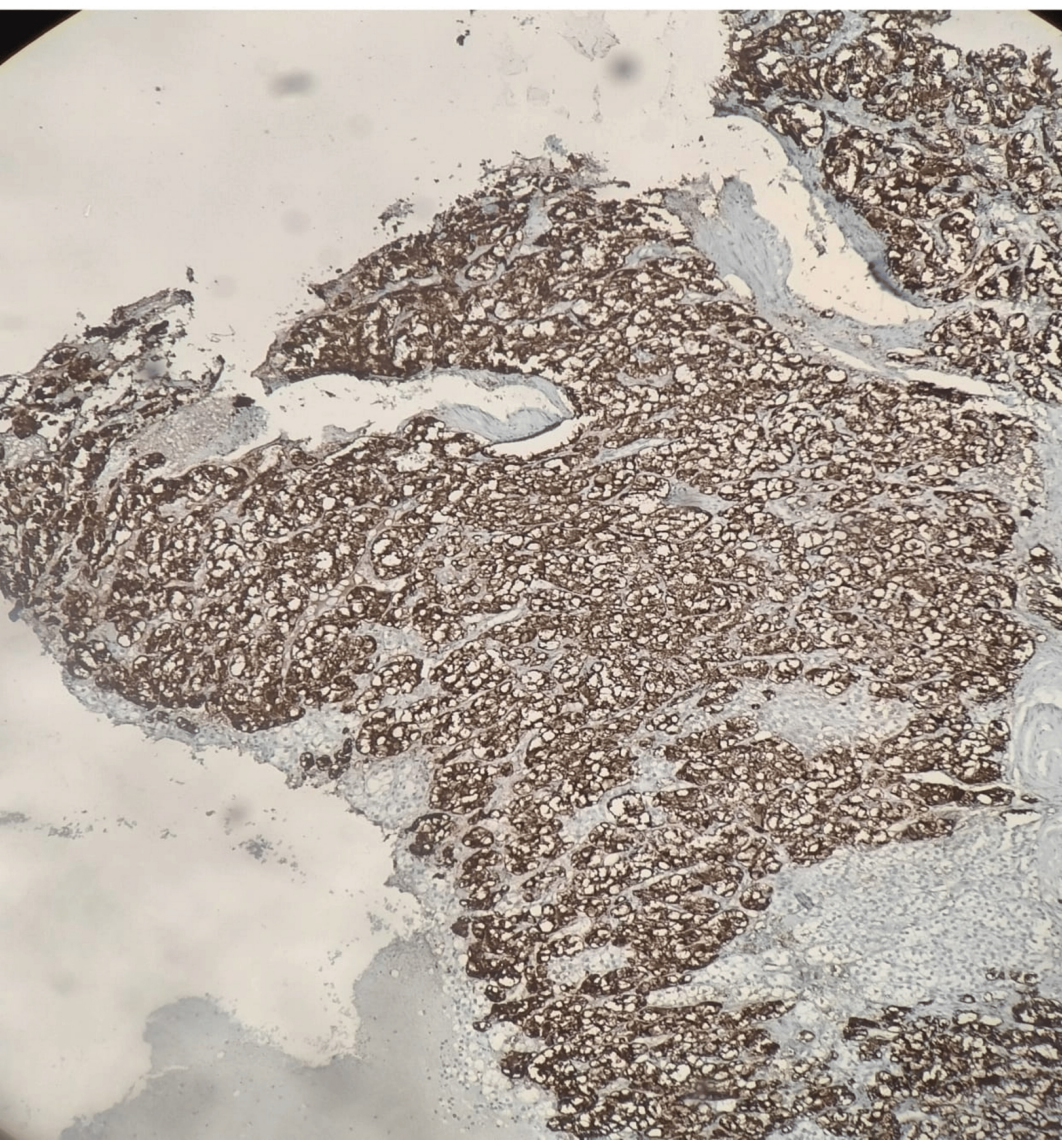
Neoplastic cells showing strong and diffuse positivity for chromogranin A suggestive of pheochromocytoma

Neoplastic cells were strongly and diffusely positive for chromogranin A (Figure 6).

**Figure 7:**
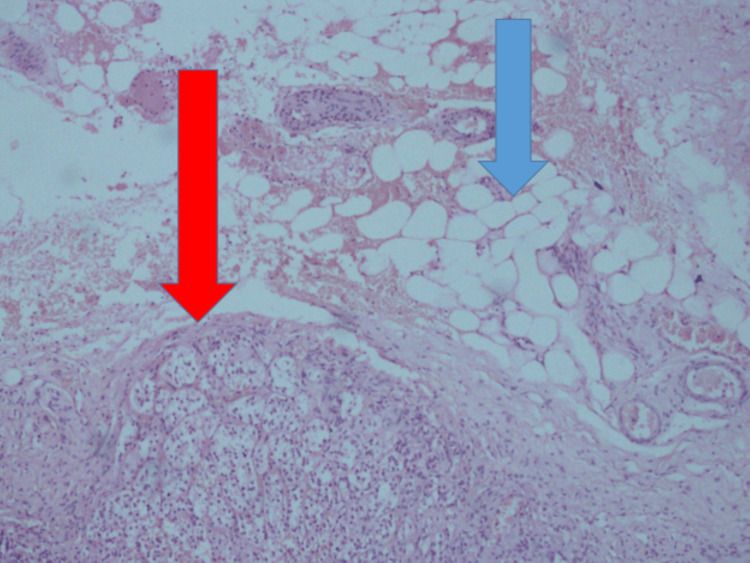
Pheochromocytoma (red arrow) at the interface with abundant periadrenal adipose tissue (blue arrow)

The pheochromocytoma focus is seen at the interface with abundant periadrenal adipose tissue (Figure 7).

Follow-up

At one month after surgery, her blood pressure was well controlled on telmisartan 40 mg/day. Repeat 24-hour urinary fractionated metanephrine and normetanephrine levels measured at four weeks after surgery were within normal limits. At three months of follow-up, prednisolone was converted to oral hydrocortisone 15 mg at 8 AM and 5 mg at 4 PM. She was evaluated for hypothalamic-pituitary-adrenal (HPA) axis recovery at three months after stopping hydrocortisone for 24 hours. However, serum cortisol remained low and was unstimulable with cosyntropin. Hydrocortisone was reinstituted, and the HPA axis was reassessed at six months. At that time, serum cortisol was normal (8.5 µg/dL), and with cosyntropin stimulation, serum cortisol was 19 µg/dL. Hydrocortisone was then stopped, and she continued antihypertensive medications. She was hemodynamically stable at her last follow-up.

## Discussion

The coexistence of pheochromocytoma with cortisol hypersecretion is exceedingly rare and diagnostically challenging, particularly when encountered in an adrenal incidentaloma. While most pheochromocytomas associated with Cushing’s syndrome have been linked to ectopic secretion of ACTH or, less frequently, corticotropin-releasing hormone (CRH), ACTH-independent cortisol secretion remains exceptional, with a limited number of biochemically confirmed cases described to date [5].

Radiological evaluation, which underpins the assessment of adrenal incidentalomas, can be misleading in this context. The 2016 ESE/ENSAT guideline advised universal biochemical screening for pheochromocytoma in patients with adrenal incidentalomas, irrespective of CT attenuation [2]. In contrast, the 2023 ESE/ENSAT guideline recommended measuring plasma-free metanephrines or urinary fractionated metanephrines in all patients with adrenal lesions that are not typical of benign adrenal adenoma [3]. Pheochromocytomas presenting with CT attenuation of <10 HU are exceedingly rare. Buitenwerf and colleagues evaluated 222 pheochromocytomas and found that only one tumor (0.5%) measured ≤10 HU. Similarly, Canu and colleagues analyzed 376 pheochromocytomas and observed none with HU <10 [8,9]. These data support that lipid-rich pheochromocytoma is very uncommon. In our patient, the overall lesion showed an unenhanced attenuation of −13 HU, strongly suggestive of a lipid-rich adenoma. However, histopathology demonstrated dual pathology with an adrenocortical adenoma and a focal pheochromocytoma component adjacent to abundant adipose tissue, indicating that the low attenuation on imaging was driven by the dominant cortical adenoma component rather than the pheochromocytoma focus itself.

The mechanisms underlying cortisol secretion in association with pheochromocytoma are varied. Cope et al. described the coexistence of pheochromocytoma and an ipsilateral adrenocortical adenoma, establishing the concept of a collision tumor [10]. Bisceglia et al. described a pigmented cortical adenoma with medullary nodular hyperplasia [11], while Sakai et al. reported a cortisol-secreting adenoma coexisting with a micro-pheochromocytoma [12]. These observations support the morphological plausibility of mixed corticomedullary tumors, although they often lacked modern biochemical documentation. More recently, Winter et al. reported an incidentaloma with dual secretion in which systemic ACTH was suppressed, but ACTH-positive cortical cells were demonstrated adjacent to the tumor, implicating intra-adrenal paracrine ACTH stimulation [5]. Similarly, several authors have documented suppressed ACTH and cortisol autonomy in patients with incidentalomas containing pheochromocytoma, confirming a distinct ACTH-independent phenotype [6,7,13].

Our patient most likely represents a collision tumor, given the presence of two separate components. Suppressed ACTH and low DHEAS provided clear evidence of ACTH-independent cortisol secretion. The exceptionally low attenuation on unenhanced CT further underscores the diagnostic challenge and highlights the diagnostic dilemma. The focal pheochromocytoma component adjacent to abundant adipose tissue was probably responsible for the low attenuation HU values on imaging. Clinically, the coexistence of catecholamine and cortisol excess has important implications, as both hormones independently contribute to hypertension, diabetes, osteoporosis, and cardiovascular morbidity. In this case, baseline hypertension and osteoporosis likely reflected these combined effects. Perioperative management required both alpha-adrenergic blockade for pheochromocytoma and perioperative glucocorticoid coverage for cortisol autonomy, with postoperative glucocorticoid replacement required due to suppression of the contralateral adrenal gland.

## Conclusions

Adrenal incidentalomas with dual hormone secretion remain a diagnostic and perioperative challenge because imaging may appear reassuring while clinically important hormonal excess is still present. A structured biochemical approach, therefore, is essential to avoid missed diagnoses and prevent avoidable complications. In our case, evidence of mild ACTH-independent cortisol secretion along with elevated urinary normetanephrine was a key pointer to dual pathology despite a very low unenhanced CT attenuation. Recognition of this pattern is important for preoperative and postoperative management of such cases.
